# Rapid and accurate analysis of stem cell-derived extracellular vesicles with super resolution microscopy and live imaging

**DOI:** 10.1016/j.bbamcr.2018.09.008

**Published:** 2018-12

**Authors:** Zubair Nizamudeen, Robert Markus, Rhys Lodge, Christopher Parmenter, Mark Platt, Lisa Chakrabarti, Virginie Sottile

**Affiliations:** a Wolfson STEM Centre, Division of Cancer & Stem Cells, School of Medicine, University of Nottingham, UK; b School of Life Sciences, University of Nottingham, UK; c School of Chemistry, University of Nottingham, UK; d Nanoscale and Microscale Research Centre, University of Nottingham, UK; e Department of Chemistry, Loughborough University, UK; f School of Veterinary Science, University of Nottingham, UK

**Keywords:** Extracellular vesicle, Super-resolution microscopy, Stem cell

## Abstract

Extracellular vesicles (EVs) have prevalent roles in cancer biology and regenerative medicine. Conventional techniques for characterising EVs including electron microscopy (EM), nanoparticle tracking analysis (NTA) and tuneable resistive pulse sensing (TRPS), have been reported to produce high variability in particle count (EM) and poor sensitivity in detecting EVs below 50 nm in size (NTA and TRPS), making accurate and unbiased EV analysis technically challenging. This study introduces direct stochastic optical reconstruction microscopy (d-STORM) as an efficient and reliable characterisation approach for stem cell-derived EVs. Using a photo-switchable lipid dye, d-STORM imaging enabled rapid detection of EVs down to 20–30 nm in size with higher sensitivity and lower variability compared to EM, NTA and TRPS techniques. Imaging of EV uptake by live stem cells in culture further confirmed the potential of this approach for downstream cell biology applications and for the analysis of vesicle-based cell-cell communication.

## Highlights

• Direct Stochastic Optical Reconstruction Microscopy (d-STORM) can be used to characterise Extracellular Vesicles (EVs).
• d-STORM imaging can detect EVs below 50 nm in size.
• The d-STORM approach results in highly reproducible EV size and concentration measurements.
• DiD-labelled EVs allow for live analysis of EV trafficking.

## Graphical abstract

**Figure:**
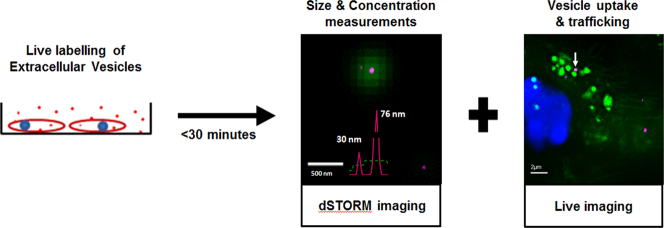

## 1 Introduction

Extracellular vesicles (EVs) are lipid membraned nanostructures secreted by cells either directly from the plasma membrane or via the endocytic pathway [1]. EVs contain and transport miRNAs [2], mRNAs [2] and active proteins [3] reported to modulate inter-cellular communication, with increased prevalence in a range of biological processes linked to cancer [4], neuroscience [5], and stem cell biology [6]. Stem cells have been reported to secrete paracrine factors largely via EVs, with relevance to immune modulation [7] and tissue repair [6]. In particular, mesenchymal stem cells (MSCs) are known to be a rich source of EVs suggested to promote healing in cutaneous wounds [8], bone fractures [9] and liver injury [10]. These observations indicate stem cells may provide a source of therapeutically useful EVs that could offer possible cell-free treatment strategies for regenerative therapy.

Cell-secreted EVs show a high degree of heterogeneity in size with apoptotic bodies and microvesicles ranging from 50 to 1000 nm, and exosomes ranging from 30 to 100 nm [1]. Exosomes can be separated into different size groups, with distinct mRNA and protein composition, and different effects on the gene expression of recipient cells [11]. It has recently been shown that EVs of 30 nm to 60 nm in size are more readily taken up by recipient cells within a 24-hour time period compared to larger EVs of 80 to 100 nm in size, resulting in higher motility of cells [12]. These recent reports highlight the importance of size as a differentiating factor for EV populations, underlining the analysis of particle size distribution (PSD) as a crucial parameter to characterise the structural and functional properties of EVs in cell biology.

Electron microscopy (EM), including scanning EM (SEM) and transmission EM (TEM), have emerged as standard techniques for EV characterisation, allowing high resolution imaging for the acquisition of size and morphology information [13], and immuno-labelling of samples to detect protein content [14,15]. Although the development of cryo-TEM has improved the preservation of sample structure and morphology [16], the uneven and inconsistent distribution of EVs onto EM grids makes it technically challenging to accurately measure concentrations. Two common alternatives to EM used to characterise EVs include nanoparticle tracking analysis (NTA) [17], and tuneable resistive pulse sensing (TRPS) [18]. However, the highly polydispersed nature of EVs make them challenging to measure using these techniques. For NTA and TRPS the minimal size range for EV detection is on average 70–150 nm, which excludes most exosomes [19]. The ability of NTA to accurately resolve two EVs, which depends on the light scattered by particles in Brownian motion [20], requires a 1.5 fold difference in their size [21], while its Raleigh approximation-based concentration estimation is strictly dependant on their refractive index, which varies with size and cargo [17,20]. By contrast, TRPS can provide more accurate size and concentration measurements by detecting current blockage from particles passing through a nanopore [22], but the detection of EVs <100 nm is problematic due to nanopore blockage by larger EVs [23]. Therefore, these techniques have significant limitations including the need for several detection settings and calibration beads for NTA [20], and the application of multiple nanopore sizes to minimise blockage in TRPS [24]. Immunoblotting (e.g. western or dot blot) is generally performed alongside these analyses to confirm the presence of EVs based on their protein content. [14,25] However, the high heterogeneity in cargos exposes protein-based quantification of EVs to inaccuracy. For instance, CD63 has been observed to be inconsistently expressed in EVs isolated from different human prostate and breast cell lines [26], while other EV markers have been found to be unevenly enriched in different proportions depending on PSD [11]. This implies that in the absence of ubiquitous EV protein markers, immunodetection approaches are inaccurate and likely to misestimate the concentration of EVs present in cell samples.

Stochastic optical reconstruction microscopy (STORM) and direct-STORM (d-STORM) are emergent single-molecule super-resolution imaging techniques with a practical resolution limit of 20 nm [27]. STORM exploits the photoswitchable property of certain fluorescent probes to localise events with high precision, and reconstruct the acquired image at a high spatial resolution [28]. As a result, STORM has been used extensively to image and characterise subcellular structures with regards to their anatomy [29], organisation [30], and biomechanical [31] properties at the nanoscale. Recently, cancer cell-derived EVs labelled using AlexaFluor 647-conjugated anti-CD63 antibodies have been imaged at high resolution using STORM [32]. Since all EVs are lipid membraned structures [33], lipophilic dyes can provide a helpful alternative to label EVs [34], irrespective of the variability in their protein content. Dyes such as Dil and its derivatives exhibit photoswitching behaviour, shifting between brightly fluorescent (light) and dark states, which enables STORM imaging of lipid-based cellular structures including the plasma membrane and lysosomes [35]. Building on this, the present investigation sought to exploit d-STORM imaging using a variant of Dil to explore the possible labelling and direct characterisation of EVs released by stem cells as a powerful alternative to existing approaches for the study of EV trafficking.

## 2 Materials and methods

All materials were purchased from Thermofisher Scientific (UK) unless stated otherwise.

### 2.1 Cell culture

Mouse mesenchymal stem cells (MSCs, D1, ATCC CRL-12424) were cultured in low-glucose Dulbecco's modified Eagle's medium (DMEM) supplemented with 10% fetal bovine serum (FBS), 1% penicillin/streptomycin (P/S), 1% l-glutamine and 1% non-essential amino acids (NEAA). 0.25% trypsin-EDTA was used for splitting the cells. Primary mouse neural stem cells (NSCs) were isolated from adult mouse lateral ventricle tissue as previously described [36] and cultured in NSC medium (DMEM F12/Neurobasal (1:1) medium containing 0.5% (P/S) and 0.01% heparin, with B27 and N2 supplements, and bFGF and EGF (both 20 ng/μl)). Accutase (Sigma) was used to split NSCs.

### 2.2 EV isolation

Prior to EV isolation, MSCs were washed with Phosphate-buffered saline (PBS) and then incubated in with EV enrichment (Exo-E) medium - containing phenol red-free low-glucose DMEM,1% l-glutamine, 1% NEAA, 1% P/S and 10% Exo-free FBS (System Biosciences), with added DiD Vybrant Cell labelling solution according to manufacturer's instructions (5 μl/ml). After 6 h, the medium was collected and filtered using 0.45 μm syringe filters (SLS). For EV isolation, exoEasy Maxi Kit (Qiagen) was used according to manufacturer's instructions. For negative controls, freshly prepared [DiD in PBS], [DiD in Exo-E medium] and [DiD in serum-free Exo-E medium], were processed in the same way. Eluted EVs were either immediately diluted and used for size distribution and particle count analysis, or stored at −80 °C for use in cell culture experiments. Samples were sonicated prior to use using Bioruptor (Diagenode) at low power three times for 10 s.

### 2.3 TEM and cryo-TEM

For TEM, samples were prepared according to a published protocol with slight modifications [14]. Briefly, samples were fixed with 4% paraformaldehyde and added (5 μl/grid) to glow discharged (10 s at 5 mA using an Agar turbo coater aux power unit and dedicated glow discharge head) Formvar‑carbon coated EM grids (EM resolutions), and adsorbed for 20 min. Sample grids were washed with PBS and incubated with 1% glutaraldehyde for 5 min, washed with sterile distilled water, and incubated with 3% uranyl-acetate for 15 min for negative staining. TEM was carried out using a Tecnai Biotwin-12 with an accelerating voltage of 100 kV. For cryo-TEM, glow discharged Holey carbon copper TEM grids were used (EM resolutions). Samples were let to adsorb onto the grids (5 μl/grid) for 20 min before the excess solution was removed using filter paper and the grids allowed drying under ambient conditions. Samples were then frozen using a Gatan CP3 plunge freezing unit, blotting for 1 s and freezing in liquid ethane. Samples were transferred to cryo-TEM storage boxes and then loaded into a Gatan 626 cryo-TEM holder on a JEOL 2100+ TEM. Images were acquired for 2–4 s at a dose of below 10 e/A^2^, using a US1000 CCD camera and Digital Micrograph GMS 3.

### 2.4 Dot blot immunodetection

MSCs and MSC-derived EV extracts were lysed using RIPA lysis and extraction buffer with added proteinase inhibitor and phosphatase inhibitor cocktails (Sigma). Protein concentration was measured using a Bradford assay (Sigma). For dot blot analysis [37], 10 μg of protein from each sample were added onto nitrocellulose membranes and dried for 10 min. The membranes were blocked using 1% skimmed milk in TBS-T (0.1% Tween20 (Sigma) in Tris buffer) and incubated with primary antibodies against CD63, TSG101, or GM130 (Santa Cruz) for 30 min. Membranes were then washed with TBS-T, incubated with peroxidase conjugated secondary antibody (vector laboratories) for 30 min, and then washed with TBS-T, before a 1-minute incubation with ECL detection reagent. Membranes were immediately imaged using LAS-4000 (Fujifilm).

### 2.5 d-STORM characterisation

For d-STORM imaging, a 1 in 1000 dilution of DiD-labelled EVs in PBS was seeded onto poly-l-lysine (Sigma) coated 4-well glass bottom Petri dishes (Greiner-Bio). Imaging was performed using a Zeiss Elyra PS1 super resolution microscope, with an α-Plan Apo 100×/1.46 oil immersion objective in TIRF (Total internal reflection microscopy) mode. Before imaging, 30 °C oil (Zeiss, Immersol™ 518F/30°) droplet was placed on 100× objectives. The LP 650filter was used to visualise EVs. TIRF was used to visualise and scan the EVs bound to the cover glass, automatic focusing (definite focus) maintained the desired focal plane during the experiment. Before the STORM experiment, a wide-field snapshot was captured using the ‘16 Avg’ option (Zeiss) at 0.5% laser power (0.02 kW/cm^2^) in TIRF mode to detect the distribution of DiD positive particles prior to d-STORM imaging. This feature was off when running d-STORM. d-STORM was run at 8% (0.31 kW/cm^2^) laser power, recording 10,000 image frames (or cycles) with 25 ms exposure per frame, and 200 gain on the EM-CCD (Andor EM-CCD camera iXon Du 897). Test runs with higher power laser and longer scans did not provide extra information for this experiment. Notably, higher laser power may damage the smallest vesicles and compromise reliable STORM analysis. An 80-second video showing the photoswitching property of DiD-labelled EVs in TIRF mode during d-STORM imaging was recorded (25 frames per second).

**Supplementary Fig. 1:**
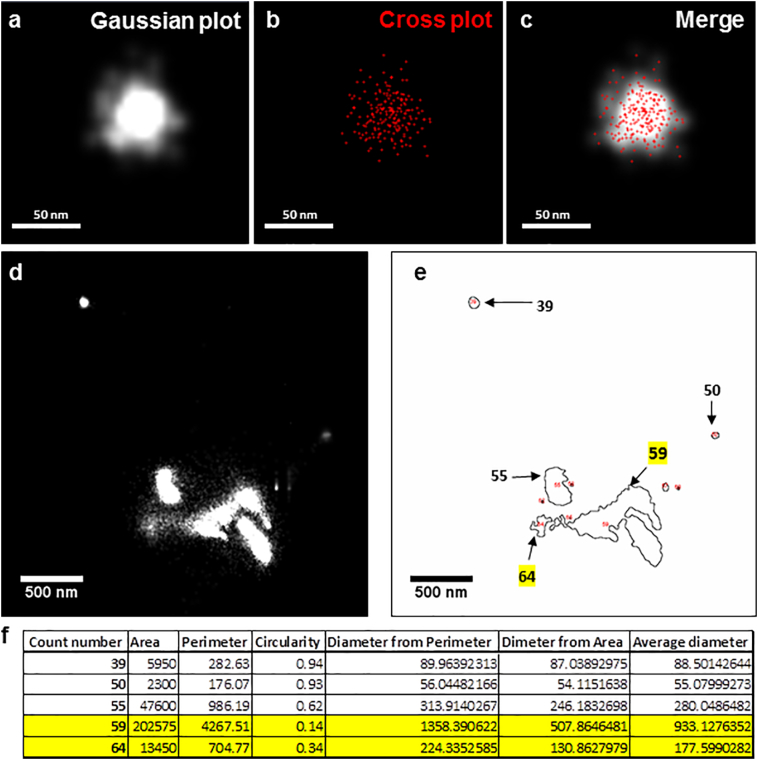
Image analysis of EVs captured using d-STORM super resolution microscopy. (a–c) Snapshots describing the precision of d-STORM gaussian plot. (a) Gaussian rendered image of a vesicular structure (white). (b) Cross plot (red) of localised molecules used to render gaussian image. (c) Merged image of gaussian and cross plot. (d–f) Snapshots describing the ImageJ analysis of exported d-STORM images. (d) Exported d-STORM image. (e) ImageJ macro mapping of vesicle shape and size. (f) ImageJ export of particle analysis. Yellow highlights point out the structures with circularity below 0.5.

Images were processed using the PALM module of the Zeiss Zen Black software. Data were filtered as follows: number of photons threshold was adjusted to 200 according to measurements made on the negative controls -PBS only and medium only (no FBS). Localisation point centroids were blurred with a Gaussian filter, whose diameter equals the localisation precision for each localised molecule. The accuracy of the rendered image can be adjusted using the expansion factor slider in the Zeiss Zen Software (point spread function, PSF). Values lower than 1 PSF render higher precision to Gaussian representation of the localised molecules. With an expansion factor of 0.25 PSF, the cluster of localisations matched the rendered image (checked using cross plots) so that vesicle sizes were not over estimated (Supplementary Fig. 1a–c). Calibration experiments were done with known size fluorescent beads (100 nm and 190 nm). To export, image resolution was set at 5 nm/pixel with Render auto dynamic range of 90%. For line profiling, EV fluorescence intensity was measured on 16-bit tiff files exported as raw data from the czi files. For comparison of resolution, images were exported as tiff files and merged with reconstructed standard wide field (rSWF, for further info please see Zeiss Zen 2012, Palm Module, Help menu) of corresponding counterparts.

An ImageJ macro was developed to identify and measure vesicles from exported 16-bit tiff files (Supplementary file 1). The processing and analysis was carried out following the steps: (i) raw tiff images exported from Zen were imported into FIJI, (ii) a Gaussian blur filter (3 pixel) was applied to the image to smooth the edges of the vesicles, (iii) the default thresholding method was used to distinguish the vesicles from the background- thresholding setting a minimum and maximum pixel intensity range on the selected image that groups all pixels falling within this range and excluding the background, (iv) Once the vesicles were identified using the thresholding tool, particle analysis was carried out to generate size, intensity, shape measurements including the area, perimeter and circularity of the particle, (v) Outline of the identified vesicles was automatically exported as tiff so that it could be compared to the original images, (vi) Results were automatically exported as a text file. Result files generated from ImageJ macro were opened in Microsoft Excel software for further analysis as follows- (i) the diameter of objects was calculated by averaging the diameter calculated from the measured area and the measured perimeter of the object, (ii) the objects were then sorted according to their circularity. Objects below a circularity of 0.5 were discarded from the dataset as they largely corresponded to aggregate or tubular like structures (Supplementary Fig. 1d–f). (iii) The resulting data set from each sample was used to create a histogram frequency set in Excel. (iv) the concentration of particles was calculated using the following equation: *Concentration* = *n* ∗ *dA* ∗ *df*, where *n* is the number of vesicles observed per imaged area, dA is the area of the dish where sample is loaded and df is the dilution factor of loaded sample, and (v) mean ± SEM was plotted using GraphPad Prism Software (https://www.graphpad.com).

### 2.6 Tuneable resistive pulse sensing (TRPS)

TRPS was performed using the qNano system (IZON Sciences, New Zealand) with the IZON Control Suite software (V3.1.2.53). NP100, NP200 or NP300 elastomeric tuneable nanopores were used, suitable for analysing beads between 85 and 600 nm (as stated by the manufacturer). Carboxylated polystyrene beads, denoted as CPC200 (Bangs Laboratories, USA), with a mean nominal diameter of 210 nm and stock concentration of 1 × 10^12^ particles/ml, were used as a concentration calibrant at 2 × 10^9^/ml. Prior to use, the beads were vortexed for 30 s and sonicated for 1 min to ensure mono-dispersity. An appropriate stretch and a voltage was applied throughout so that the blockades of CPC200s in PBS were at least 0.5 nA above the background noise. The qNano was operated as previously described [38]. Briefly, the lower fluid cell was filled with 75 μl of PBS, ensuring no air bubbles are present and the upper fluid cell contained 40 μl of sample. After each measurement, the sample was removed from the upper fluid cell and replaced with PBS. This was repeated several times, applying varying amounts of pressure and vacuum, until visible blockades were observed.

### 2.7 Nanoparticle tracking analysis (NTA)

A LM10/14 Nanosight (Nanosight, Malvern) instrument was used to analyse EVs. Prior to analysis, 1:10 dilution of CPC100 (IZON) and 1:1000 dilution of 200 nm polystyrene (Malvern) nanoparticles were used to test the sensitivity of the instrument. EV samples were used at 1:500 dilution. Automatic settings were applied for the minimum expected particle size, minimum track length and blur settings. For capture settings, screen gain was set at 1 and camera level was set at 10 (shutter 1500; gain 680). For analysis settings, screen gain was set at 10 and detection threshold was set at 10. Five movies of 60 s were captured at 30 frames per second for each sample. Data processing and analysis of particle size distribution and concentration were performed using NTA Software (https://www.malvern.com). NTA concentration estimation is dependent on the refractive index of particles under analysis according to the Rayleigh approximation $\sigma_{s}=\frac{2\pi^{5}}{3}\frac{d^{6}}{\lambda^{4}}{\left(\frac{n^{2}-1}{n^{2}+2}\right)}^{2}$ (where d is the particle diameter, λ is the wavelength, and n is the ratio of particle refractive index to solvent refractive index [17]), which is known to vary in EV samples due to heterogenic size and content [11]. Therefore, NTA analysis was used only to determine PSD but not EV concentration.

### 2.8 Confocal microscopy, structured illumination microscopy (SIM) and real-time wide-field imaging

NSCs were seeded at 1 × 10^5^ cells/cm^2^ and left to grow for 24 h. Vybrant DiO was used to stain NSCs in culture according to manufacturer's protocol. NSCs were then incubated with 5 × 10^8^ DiD labelled MSC derived-EVs as calculated by d-STORM. Nuclei were labelled with Hoechst 33258 according to manufacturer's instructions. Samples were imaged within 30 min. For confocal microscopy, real-time wide-field imaging and structured illumination microscopy (SIM), Zeiss Elyra PS.1 microscope equipped with C-Apochromat 63×/1.2 W Korr M27 objective was used. Lasers 633 (10%), 488 (0.2%) and 405 (2%) were used for confocal imaging. A pinhole of 1.06 Airy unit was used to image the full field of view, to have an optical slice equivalent of 1 μm thickness. For real-time wide-field imaging, the lasers 642 (1%), 488 (0.02%) and 405 (2%) were used with multi-bandpass filter BP 420–480 + BP 495–550 + LP 650 at exposure time of 40 ms and 200 camera gain on EMCCD camera (25 frames per second). The microscope had access to ‘internal’ hardware switch option which was used for fast imaging. For SIM, the following settings were used: multi-bandpass filter set BP 420–480 + BP 495–550 + LP 650, camera exposure time 35.0 ms, lasers 642 (20%), 488 (8%), 405 (8%), grating period 51 μm.

### 2.9 Statistical analysis

All experiments were run in triplicates using separate cell culture preparations (biological replicates), with at least three internal repeats (technical replicates). Mean and SEM were analysed using GraphPad Prism Software and Microsoft Excel (unless specified otherwise). One-way ANOVA with multiple comparison tests was run to determine the statistical significance for all analyses unless mentioned otherwise. Statistical significance levels were set for *p < 0.05, **p < 0.01, ***p < 0.001, ****p < 0.0001. All graphs were made using GraphPad Prism Software (https://www.graphpad.com).

## 3 Results

### 3.1 Sample analysis of stem cell-derived EVs by TEM, cryo-TEM and immuno-dot blot

**Fig. 1:**
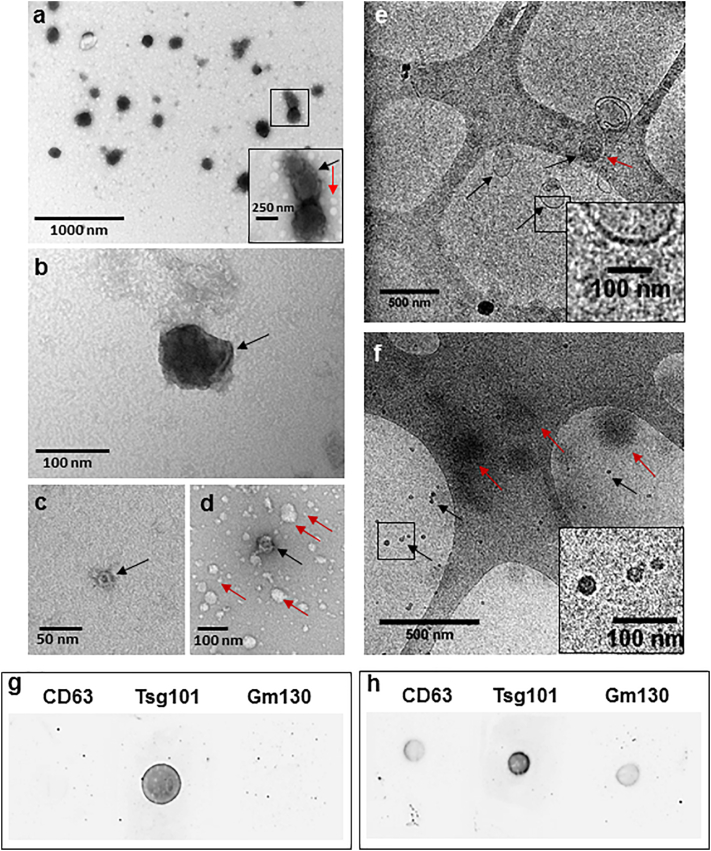
Characterisation of EVs isolated from MSC culture supernatant. (a–d) TEM images of EVs. (a) TEM showing polydispersity EV structures of 100–250 nm EVs. Zoomed-in box shows cup-shaped morphology of a vesicle (black arrow) and non-cup-shaped white vesicular structures below 50 nm in size (red arrow). (b) 100 nm vesicle, black arrow showing membrane fold. (c) 20 nm cup-shaped EV (black arrow). (d) 50 nm cup-shaped EV structure (black arrow) along with other white, non-cup-shaped vesicular structures of 5 to 100 nm in size (red arrows). (e and f) Cryo-TEM images of EVs. (e) Image showing spherical (black arrows) and elongated (red arrow) EV structure with a clear membrane (zoomed-in box). (f) Images showing 20 nm EVs with defined borders (black arrows and zoomed-in box) and larger EVs with undefined borders (red arrows). Scale bars are defined in each figure. (g–h) Dot blot immunodetection of CD63, TSG101 and GM130 proteins in (g) EV lysate and (h) cell lysate samples isolated from MSCs.

In addition to producing a variety of paracrine factors involved in tissue regeneration, MSCs produce a large number of EVs that can be collected in vitro. To identify the structure and morphology of EVs released from undifferentiated MSCs, TEM and cryo-TEM were performed (Fig. 1). TEM images confirmed the presence of polydispersed EV structures from 20 to 250 nm in size (Fig. 1a-d). The identified EVs showed cup shaped morphology (Fig. 1a) and membrane folds (Fig. 1b) similar to previously published reports [25]. Smaller EVs from 20 to 50 nm in size were also identified (Fig. 1c,d). Spherical structures from 5 to 100 nm in sizes were also identified that lacked a cup shaped morphology and contrast (Fig. 1a,d) which could correspond to the presence of lipoprotein vesicles as suggested by previously published reports [39]. This suggested that the sample contained EVs of 20 to 250 nm in size along with other lipid vesicular structures of 5 to 100 nm in size.

As cryo-TEM avoids fixation and dehydration used for conventional TEM procedures, fresh samples were analysed by cryo-TEM to visualise EVs with minimal damage (Fig. 1e,f). This revealed EVs from 20 to 250 nm in size presenting round morphologies (Fig. 1e–f). Larger EVs of 150 to 250 nm in sizes with clear membrane borders with some of them having elongated membranes (Fig. 1e) similar to previously published reports [16], were observed in low numbers. 20 to 50 nm structures were found in higher numbers with better overall contrast suggesting the presence of electron-dense cargo associated with them (Fig. 1f). Many structures with spherical shape but undefined borders were also found (Fig. 1f) which could suggest presence of lipoprotein structures and other contaminating proteins [16]. However, cryo-TEM images tended to provide poor contrast, and uneven vesicle distribution on EM grids resulted in lengthier imaging time and inconsistent particle counts for both TEM techniques.

To confirm the nature of the material in the samples, immunodetection was used to test for the presence of markers known to be enriched in exosomes (Fig. 1g,h) such as TSG101, a member of endosomal sorting complex required for transport (ESCRT)-related proteins, and the tetraspanin CD63 [40]. Dot plots prepared from EV samples showed positive signal for TSG101 but not CD63 (Fig. 1g), suggesting that MSC-derived EVs may lack CD63 protein. The absence of signal for the Golgi protein GM130 (Fig. 1g,h), found in cells but not in exosomes, confirmed the purity of the EV sample.

### 3.2 Direct stochastic optical reconstruction microscopy (d-STORM) imaging of stem cell-derived EVs

**Fig. 2:**
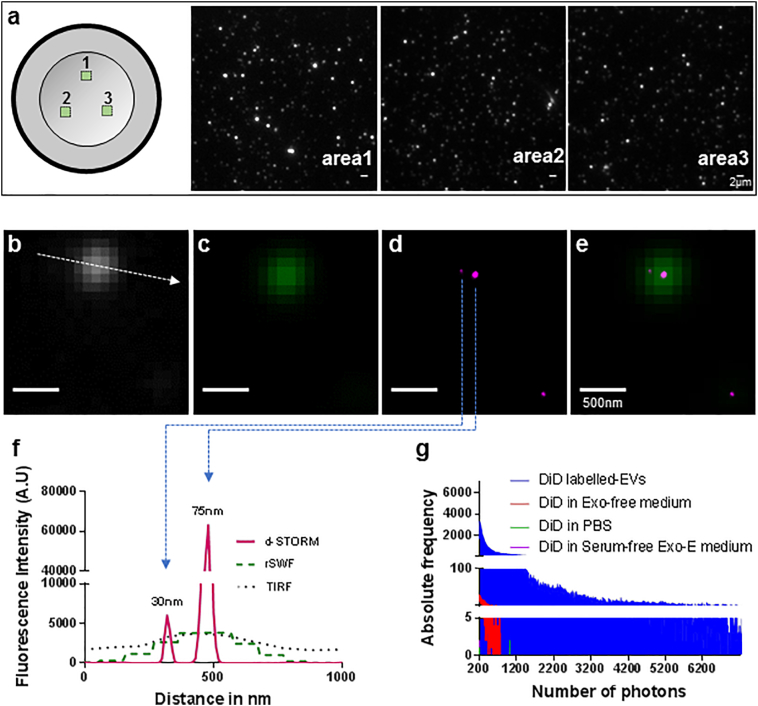
d-STORM imaging of DiD-labelled EVs isolated from MSCs. (a) Graphical representation of the sample scanning pattern with snapshots of areas 1, 2 and 3 recorded in TIRF. Scale bar: 2 μm. (b–f) d-STORM images of MSC-derived EVs showing (b) TIRF (white), (c) rSWF (sum of wide field frames over the experiment) (green 75), (d) d-STORM (magenta) and (e) merge of rSWF and d-STORM. Scale bar: 500 nm. (f) Cross-sectional line-profiling of 2 EVs imaged in TIRF, rSWF and d-STORM as shown in (b) to (e), measured across the line indicated as dotted arrow in (b). (g) Absolute frequency of photon number recorded in [DiD in PBS] (green), [DiD in Exo-E medium] (red), [DiD in serum-free Exo-E medium] (purple) and DiD-labelled EV sample (blue).

**Supplementary Fig. 2:**
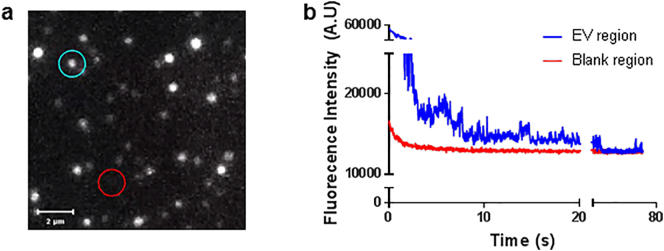
Photo-blinking frequency of DiD-labelled EVs isolated from MSCs during d-STORM imaging. (a) Screenshot from the video used to calculate the photo-blinking frequency of DiD-labelled EVs during STORM imaging, showing a positive blinking region with EVs (blue circle) and a background region for blank reading (red circle). (b) Graphical representation of the photo-blinking frequency of DiD-labelled EVs (blue) against blank region (red).

Super-resolution fluorescence microscopy techniques have become widely used to visualise cellular nanostructures [35]. d-STORM imaging in particular is known to resolve subcellular structures up to 20 nm in size [41], and would therefore theoretically allow resolution of EVs. To test this point, cultures of undifferentiated MSCs were incubated with EV enrichment medium (Exo-E) containing vybrant-DiD to label all lipid structures including EVs; these were then isolated from the medium and seeded onto poly-l-lysine charged dishes. The positively charged coating was used to promote the settlement of negatively charged EVs and thus decrease their mobility in order to facilitate d-STORM imaging [32]. DiD-labelled EVs showed a high frequency of single molecule blinking events (Supplementary Fig. 2 and Supplementary video 1), which confirmed the photoswitchable property of the DiD dye to light and dark states. Contrary to EM observations, EVs were distributed evenly throughout the dish, allowing for rapid imaging of multiple vesicles in every field of view (Fig. 2a). d-STORM imaging was able to resolve structures <200 nm apart and detect EVs of 20–30 nm in size (Fig. 2b–f), which was comparable to earlier results from TEM in terms of spatial resolution. Moreover, EVs larger than 250 nm in size were rarely observed in MSC-derived samples, which was consistent with the cryo-TEM observations.

To confirm the d-STORM imaging results and exclude possible artefacts, EV-free samples ([DiD in PBS], [DiD in Exo-E medium] and [DiD in serum-free Exo-E medium]) were analysed in parallel to evaluate non-specific fluorescence in the far-red channel (Fig. 2g). [DiD in PBS] used as control showed no photo-blinking events in the far-red channel, suggesting the absence of non-specific fluorescence from unbound dye. [DiD in Exo-E medium] produced rare single molecule events at a photon count which was 100 times lower than the experimental samples (Fig. 2g), while d-STORM imaging using [DiD in serum-free Exo-E medium] showed no detectable photo-blinking events (Fig. 2g), indicating that the weak signal observed with [DiD in Exo-E medium] was likely due to residual EVs from the serum. Collectively, the d-STORM approach generated direct and reproducible datasets of EVs released from MSC cultures observed to be in the 20–250 nm range, with no sample processing post EV isolation, resulting in minimal artefactual modification of morphological features.

### 3.3 Size and concentration characterisation of stem cell-derived EVs using d-STORM vs NTA and TRPS

**Fig. 3:**
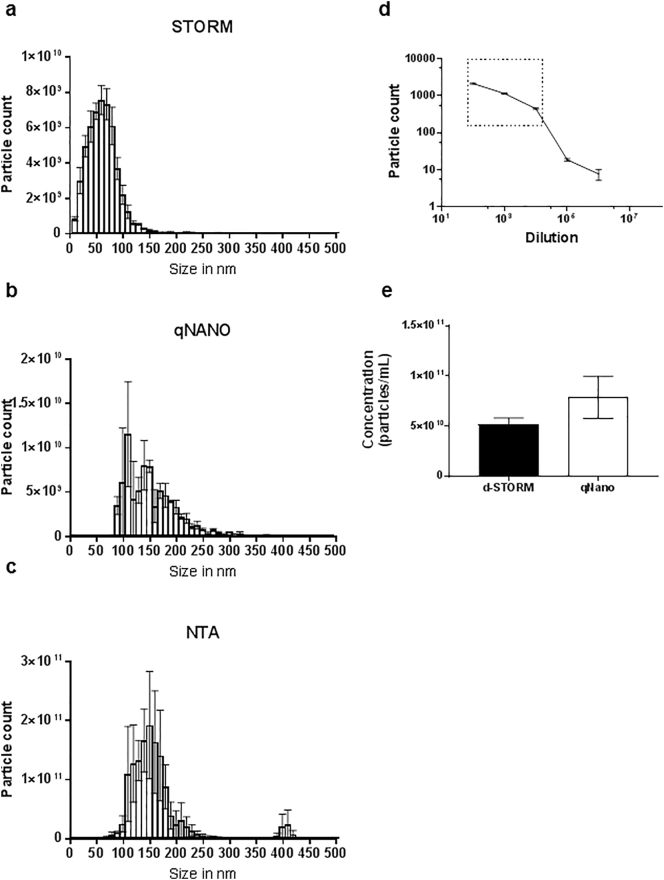
Size distribution and concentration analysis of EVs isolated from MSCs. (a–c) Particle size distribution analysed by (a) d-STORM imaging, (b) TRPS and (c) NTA. (d) Standard curve from DiD-labelled EV dilutions to determine the working range for d-STORM particle count, shown in the boxed area. (e) EV concentration measurements produced by d-STORM and TRPS based approaches. Data represented as mean ± SEM, *p < 0.05 (n = 3).

NTA [17] and TRPS [22] are the most commonly used alternative approaches to EM for the analysis of EVs, due to their requirement for comparatively limited sample processing and speed of analysis [15]. To analyse the size distribution of MSC-derived EVs, measurements using LM10/14 (NTA) and qNano (TRPS) were performed alongside the new d-STORM approach (Fig. 3), and sensitivity was compared. The size distribution obtained by d-STORM indicated a majority of EVs under 100 nm in size, with a significant proportion of EVs (ca. 41.6%) below 50 nm in size (Fig. 3a). By contrast, TRPS and NTA measurements did not detect EVs smaller than 50 nm in the samples (Fig. 3b,c). The TRPS instrument failed to maintain consistent readings for the detection of particles below 100 nm in size (Fig. 3b), as it was observed that the specific nanopore NP100 used for smaller EVs experienced repeated blockages by larger particles during the analysis. On the other hand, NTA analysis indicated a majority of EVs in the size range of 100 nm and above (Fig. 3c), in line with its reported overestimation of larger particles in highly polydispersed samples [42]. Overall, the PSD results obtained by d-STORM for MSC-derived EVs presented less variability (Fig. 3a–c) compared to NTA and TRPS results, and showed an increase in sensitivity to vesicles within the smaller size range below 50 nm.

The concentration of MSC-derived EVs was analysed as it is a critical parameter to standardise cell-based experiments based on exosomal uptake. While TRPS was used and provided a concentration of 7.86 ± 2.11 × 10^10^ particle/mL, NTA analysis was not suitable as it is dependent on the particle refractive index, which is known to vary in EV samples due to size and content heterogeneity [17,20]. In order to extrapolate concentration values from the d-STORM measurements, serial dilutions of EVs were analysed to generate a standard curve, which produced a linear range between 1:100 to 1:10000 dilutions enabling the measurement of EV concentration in MSC-derived samples (Fig. 3d). d-STORM measured a EV concentration of 5.23 ± 0.62 × 10^10^ particle/mL, with 3.4 times less variability compared to the TRPS analysis obtained for the same sample (Fig. 3e). These results established that d-STORM could be used to provide simultaneous EV size and concentration measurements, with better accuracy compared to values obtained by standard nanopore-based TRPS.

### 3.4 Imaging of EV uptake in stem cell culture using confocal, SIM and live-epifluorescence microscopy

**Fig. 4:**
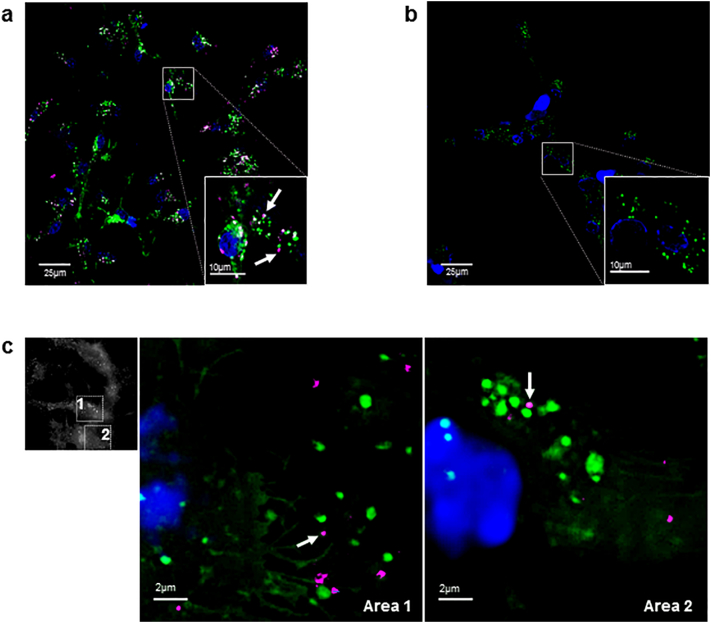
Cellular uptake of MSC-derived EVs imaged in live NSC culture. (a–b) Confocal microscopy imaging of live DiO-labelled NSCs (green) with nuclear counterstain (blue), incubated in the (a) presence or (b) absence of DiD-labelled EVs. Arrows show DiD-labelled EVs in magnified view of boxed area. (c) SIM imaging of DiD-labelled EVs (red) uptake by DiO-labelled NSCs (green), with magnified areas 1 and 2 showing intracellular DiD-labelled EVs (arrows).

To determine whether the analytical approach developed for d-STORM characterisation could be used to directly examine whether MSC-derived EVs enter live cells, NSCs labelled with vybrant DiO (DiO-NSCs, green) were incubated in the presence or absence of DiD-labelled MSC-derived EVs (DiD-EVs, red). Live confocal microscopy within 30 min of incubation detected multiple DiD-EVs in the cytoplasm of DiO-NSCs exposed to MSC-derived EVs (Fig. 4a), but not in control DiO-NSCs incubated in the absence of DiD-EVs (Fig. 4b), indicating the rapid uptake of MSC-derived EVs by NSCs.

Internalised DiD-EVs were examined using structured illumination microscopy (SIM), which provided a 2-fold increase in spatial resolution compared to wide-field microscopy, allowing visualisation of cellular structures at 100 nm resolution [43]. SIM analysis established that internalised DiD-EVs had a mean size of 230 ± 59.67 nm (Fig. 4c), in line with d-STORM and TEM results indicating EVs were below 250 nm in size. These were significantly smaller than endogenous DiO-labelled structures seen in NSCs (mean size 765 ± 123.24 nm), which match recent reports that cells contain lipid vesicles ranging from 50 to 1000 nm in size [1]. Application of real-time wide-field fluorescence imaging further documented the movement of MSC-derived EVs present within recipient NSCs alongside endogenous intracellular DiO-labelled bodies (Supplementary Video 2). This experiment established that EV exposure to the DiD labelling and storage steps employed for the d-STORM approach developed here did not interfere with their capacity for cellular uptake and trafficking in live cells.

## 4 Discussion

EVs have been attracting increasing interest in the biomedical field since their recognition as an acellular communication mechanism in stem cell and cancer biology [4], and for their efficiency as nanoparticle and drug delivery vehicles for clinical therapy [44]. Various technologies have been developed to characterise EVs in terms of size, concentration and protein content [25]. The present study shows the development of a characterisation approach based on d-STORM imaging to analyse stem cell-derived EVs collected in culture.

Previous studies have reported that MSC-derived EVs isolated via ultracentrifugation can range from 40 to 200 nm in size [3,[45], [46], [47], [48]]. Here it is shown that MSC-derived EVs isolated from culture supernatants using membrane affinity columns were within 20 to 250 nm, covering the size range of EVs reported in earlier studies. The d-STORM datasets showed MSC-secreted EVs to include a majority of small-sized exosomes of 20 to 100 nm in size, in agreement with the literature [49]. These results compared favourably with those obtained from conventional EM, NTA and TRPS measurements with respect to reliability, sensitivity and efficiency of the EV analysis. The DiD labelling approach provided key advantages, as it avoided reliance on protein-based markers, known to be inconsistent due to the heterogeneity of exosomal expression [11,26,50]. DiD-labelling provided a simple and uniform labelling of all EVs irrespective of size or cargo, widening the detection range and avoiding demanding and/or damaging sample processing steps. Importantly, DiD labelling is stable [35], compatible with storage while preserving the integrity and bioactivity of samples, and could enable multiplexing to simultaneously label other EV components such as protein or nucleotides cargo using specific auxiliary fluorophores.

Compared to conventional techniques available for EV characterisation, the present study achieved the simultaneous analysis of PSD with high sensitivity for EVs below 50 nm in size, and high-throughput particle concentration measurement. d-STORM results reliably aligned with the particle size information from cryo-TEM, while the whole procedure from sample isolation to EV characterisation was completed in under 1 h, establishing d-STORM super-resolution microscopy as an efficient and reliable alternative to EM, NTA and TRPS techniques. Interestingly, vesicular structures analysed using both TEM and d-STORM might not all be lipid bi-layered vesicles. EV-like structures without defined bilayer membrane could be lipoproteins co-isolated with typical EVs, particularly using one-step protocols such as the Qiagen exoEasy Maxi Kit used here, as these are known to exist in the size range of 5 to 1200 nm [39]. In future studies, density cushion size exclusion chromatography columns could be used to purify bi-layered EVs from lipoproteins in EV isolates for further analysis [39]. Cryo-TEM observations of processed samples in this study showed only a few vesicular structures with typical characteristics of EVs including a defined bilayer membrane analysed in recent reports [16]. This could be due to the low power sonication setting (Diagenode Bioruptor) used in this study to avoid aggregation of EVs [51,52] for d-STORM imaging, possibly disrupting EV membranes and releasing smaller vesicles and fragments able to reform smaller vesicles. Sonication of EVs has been reported to disrupt and generate pore formation in EV membranes [53], suggesting the need for a closer evaluation of membrane damage due to sonication.

The DiD dye based d-STORM approach used here was able to rapidly identify all lipid-based structures in the EV isolate and provide useful information on their shape, size and concentration. The substantial time-efficiency, sensitivity and unbiased nature of this new approach supports its potential for the next generation high-throughput EV characterisation, such as recent antibody-based microfluidics assays developed for high-throughput detection of EVs from biological fluids and cell culture media. As these are based on EV surface markers [54], they could now be coupled with the d-STORM approach to address the issue of EV heterogeneity in protein expression. The novel EV analysis modality developed here is not cell type-specific and is directly applicable to other lineages and models. In particular, it will now be useful to extend this approach to the monitoring EV populations secreted as stem cells undergo differentiation, as reports suggest that EVs can influence the differentiation status of analogous as well as different cell types [55,56].

Live imaging using super-resolution microscopy techniques have emerged as powerful means to uncover and better understand the biomechanics of subcellular events including fast reorganisation of actin cytoskeleton in macrophages and complex dynamics of bacterial filopodium movement [34,[57], [58], [59]]. However, due to long image acquisition periods, real-time super-resolution imaging of fast occurring subcellular events, including EV trafficking, has not yet been established [57]. Particularly, the use of d-STORM for live imaging of EV intracellular trafficking can be technically challenging [57,60]. The reliability of this imaging technology is based upon the precise localisation of a photoswitching event of a fluorescent molecule, which is impractical for mobile EVs in a living cell, especially if they move significantly between two localisation events. However, future adaptations of d-STORM imaging might be able to address this issue along with other advanced live-microscopy techniques including high-speed camera wide field microscopy, confocal and SIM imaging, to allow full tracking of vesicle traffic and cargo transfer using in vitro culture models.

## 5 Conclusions

d-STORM provided a quicker and more reliable approach than conventional techniques to characterise stem cell-derived EVs in one step. Extension of this d-STORM approach to investigating EV biology offers significant potential, including future developments such as multiplexing with additional labels for concomitant cargo analysis and live intracellular EV imaging to analyse EV sorting and trafficking mechanisms. This could be exploited to analyse the release and functional contribution of EVs from distinct stem cell populations, and could also be applied to the screening of EVs from cancer tissues, since EVs have been proposed as potential tumour biomarkers.

The following are the supplementary data related to this article.

Supplementary Video 1 Photo-blinking effect of DiD-labelled EVs (blue circle) against background region (red circle) during d-STORM imaging. Supplementary Video 1

Supplementary Video 2 Real-time epi-fluorescence recording showing cellular intake and active migration of MSC-derived EVs (DiD-labelled, magenta) inside live NSCs (DiO-labelled, green), with nuclear counterstain (blue). Supplementary Video 2

## Transparency document

Transparency document Image 1
